# The rs11755527 polymorphism in the *BACH2* gene and type 1 diabetes mellitus: case control study in a Brazilian population

**DOI:** 10.20945/2359-3997000000214

**Published:** 2020-03-18

**Authors:** Cristine Dieter, Natália Emerim Lemos, Luiza Emy Dorfman, Guilherme Coutinho Kullmann Duarte, Taís Silveira Assmann, Daisy Crispim

**Affiliations:** 1 Divisão de Endocrinologia Hospital de Clínicas de Porto Alegre Porto Alegre RS Brasil Divisão de Endocrinologia, Hospital de Clínicas de Porto Alegre, Porto Alegre, RS, Brasil; 2 Faculdade de Medicina Universidade Federal do Rio Grande do Sul Porto Alegre RS Brasil Faculdade de Medicina, Universidade Federal do Rio Grande do Sul (UFRGS), Porto Alegre, RS, Brasil; 3 Universidade do Vale do Rio dos Sinos São Leopoldo RS Brasil Universidade do Vale do Rio dos Sinos (Unisinos), São Leopoldo, RS, Brasil; 4 Departamento de Nutrición, Ciencia de los Alimentos y Fisiología Universidad de Navarra Navarra Espanha Departamento de Nutrición, Ciencia de los Alimentos y Fisiología, Universidad de Navarra, Navarra, Espanha

**Keywords:** Type 1 diabetes mellitus, DNA polymorphisms, *BACH2* gene

## Abstract

**Objective:**

Type 1 diabetes mellitus (T1DM) is an autoimmune disorder caused by a complex interaction between environmental and genetic risk factors. BTB domain and CNC homolog 2 (*BACH2*) gene encodes a transcription factor that acts on the differentiation and formation of B and T lymphocytes. BACH2 is also involved in the suppression of apoptosis and inflammation in pancreatic beta-cells, indicating a role for it in the development of T1DM. Therefore, the aim of this study was to evaluate the association of the BACH2 rs11755527 single nucleotide polymorphism (SNP) with T1DM.

**Subjects and methods:**

This case-control study comprised 475 patients with T1DM and 598 nondiabetic individuals. The *BACH2 rs11755527* (C/G) SNP was genotyped using real-time PCR with TaqMan MGB probes.

**Results:**

Genotype distributions of rs11755527 SNP were in accordance with frequencies predicted by the Hardy–Weinberg equilibrium in case and control groups and were similar between groups (P = 0.729). The minor allele frequency was 43.6% in cases and 42.5% in controls (P = 0.604). Moreover, the G allele frequency did not differ between groups when considering different inheritance models and adjusting for age, gender, body mass index, and *HLA DR/DQ* genotypes of high-risk for T1DM. Although, well-known high-risk T1DM *HLA DR/DQ* genotypes were associated with T1DM in our population [OR= 7.42 (95% CI 3.34 – 17.0)], this association was not influenced by the rs11755527 SNP.

**Conclusion:**

The BACH2 rs11755527 SNP seems not to be associated with T1DM in a Brazilian population.

## INTRODUCTION

Type 1 diabetes mellitus (T1DM) affects 10-15% of patients with diabetes mellitus (DM) and is caused by autoimmune destruction of pancreatic beta-cells, making patients dependent of insulin for life (1,2). This disease most likely arises from a multifaceted interaction between multiple environmental and genetic risk factors (2). The *HLA DR/DQ* locus has the greatest impact on susceptibility, accounting for up to 30-50% of the genetic variance of T1DM (3). Although more than 50 genes have been described as having smaller effects on T1DM susceptibility in comparison to *HLA* loci, it has been suggested that the interaction between *HLA* haplotypes and non-*HLA* single nucleotide polymorphisms (SNPs) could be useful to help improve prediction of the disease (4,5). Consequently, identification of non-*HLA* SNPs associated with T1DM may help disease prediction (6).

Many genome-wide association studies (GWAS) have found a number of *loci* associated with T1DM [reviewed in (4,7)], including polymorphisms in the BTB domain and CNC homolog 2 (*BACH2*) gene. *BACH2* encodes a transcription factor that acts in the immune system, which is involved in the development and function of alveolar macrophages, B cell differentiation, somatic hypermutation, class switching of immunoglobulins, maintenance of naïve T cell state, and formation of regulatory T cells (8-10).

Additionally, this gene is expressed and regulated by proinflammatory cytokines in pancreatic islets in both rodents and humans (11). *BACH2* inhibition aggravated cytokine-induced apoptosis of pancreatic beta-cells through activation of the JNK1/BIM pathway, whereas opposite effects were observed after *BACH2* overexpression. Hence, SNPs in the *BACH2* gene might contribute to development of T1DM since this gene is related to T cell control and B cell differentiation, modulating the balance between tolerance and immunity, and also plays an anti-apoptotic role in beta-cells (11).

To date, few studies have evaluated the association between *BACH2* SNPs and T1DM, mainly in North American and European populations (12-18). Further studies are therefore needed to attempt to replicate the association between *BACH2* SNPs and T1DM in different ethnicities with diverse genetic and environmental backgrounds. Here, we analyzed whether the rs11755527 SNP in the *BACH2* gene is associated with T1DM in a Southern Brazilian population of mixed ethnicity.

## SUBJECTS AND METHODS

### Samples

The STROBE and STREGA guidelines were used to design this case-control study (19,20). The case group comprised 475 T1DM patients recruited from outpatient clinic at the Hospital de Clínicas de Porto Alegre (Rio Grande do Sul, Brazil) between January 2005 and December 2013. American Diabetes Association guidelines were followed when diagnosing T1DM patients (1).

The control sample consisted of 598 nondiabetic blood donors recruited from the same hospital as the patients and over the same period. Subjects with HbA1c levels ≥ 5.7% were excluded from the control group (1). For both groups, individuals for whom data on ethnicity, *HLA* genotype or HbA1c were missing were excluded from the study. Ethnicity classification was based on self-classification. All subjects provided assent and written informed consent prior to inclusion in the study.

Data on age, age at T1DM diagnosis and drug treatment were collected using a standard questionnaire. Patients with T1DM also underwent physical examinations and laboratory tests, as previously reported by our group (21). Plasma and serum samples of T1DM patients were collected after a 12 h of fasting for laboratory analyses, as previously reported (21).

### Genotyping

A standardized salting-out technique was used to extract total DNA from peripheral blood leukocytes. The SNP rs11755527 (C/G) in the *BACH2* gene was genotyped using TaqMan SNP Genotyping Assay 20x (Thermo Fisher Scientific, Foster City, CA, USA ID Assay: C__2014214_10). Real-Time PCR reactions were run in 384-well plates, using 2 ng of DNA, TaqMan Genotyping Buffer 1x (Thermo Fisher Scientific) and TaqMan SNP Genotyping Assay 1x, in a final volume of 5 mL. Next, plates were placed in the ViiA7 Real-Time PCR System (Thermo Fisher Scientific) and heated for 10 min at 95º C, followed by 50 cycles of 95º C for 15 sec and 63º C for 90 sec. Ten percent of samples were amplified twice in order to check the quality of Real-Time PCR reactions, confirming their genotypes.

Taking into account that *HLA DR/DQ* genotypes may affect the association between non-*HLA* SNPs and T1DM (22), we also analyzed frequencies of *HLA* high-risk genotypes in all subjects in order to control for a possible association between the *BACH2* rs11755527 SNP and T1DM for the *HLA* genotypes. To achieve this, three SNPs adjacent to the *HLA class II* region (rs2854275, rs9273363 and rs3104413) were genotyped using Custom TaqMan Genotyping Assays 20x (Thermo Fisher Scientific), as previously reported (23,24). This method was used considering that Nguyen and cols. (22) showed that these SNPs can predict *HLA DR/DQ* genotypes associated with T1DM with an accuracy higher than 99%. Thereafter, using this method, we calculated frequencies of the following *HLA DR/DQ* genotypes: low-risk genotypes (*DRx/DRx* or *DR4/DQ7*), intermediate-risk genotype (*DR3/DRx*), and high-risk genotypes (*DR4/DQ8* or *DR3/DR4-DQ8* or *DR3/DR3*) (22,23). These three SNPs occur in the intergenic region between *HLA-DRB1, HLA-DQA1* and *HLA-DQB1*, and are in strong linkage disequilibrium with the *HLA* high-risk genotypes. Consequently, they have high accuracy for prediction of *HLA DR/DQ* high-risk genotypes (22).

### Statistical analyses

Deviations of genotype frequencies from the Hardy–Weinberg equilibrium (HWE) were calculated using c^2^tests. Genotype and allele frequencies were compared between case and control groups with c^2^ tests. Moreover, genotypes were also compared between groups under different inheritance models (dominant, recessive and additive) (25,26). Laboratory and clinical variables were compared between cases and controls using unpaired Student’s t or c^2^ tests. Bonferroni’s correction was used to account for multiple comparisons.

Logistic regression analyses were used to estimate the odds ratio (OR) with 95% CI and P values for the effects of *BACH2* genotypes on T1DM susceptibility, both for genotype frequencies and the different inheritance models, and adjusting for covariates. Interactions between *BACH2* and high-risk T1DM *HLA DR/DQ* genotypes were investigated using generalized linear model – GLM (binary logistic model) analysis, adjusting for age, gender, and BMI. The power calculation was performed using the WinPepi program (v. 11.65). The study has a power of 80% (a = 0.05) to detect an OR ≥ 1.4 (for the recessive model of inheritance). All analyses were performed using SPSS 18.0 software (SPSS, Chicago, IL), and P values < 0.05 were considered significant.

## RESULTS

### Sample description

The main characteristics of the subjects in case and control groups are shown in Table 1. Mean HbA1c values and frequencies of hypertension and high-risk T1DM *HLA DR/DQ* genotypes (*DR4/DQ8, DR3/DR4-DQ8* or *DR3/DR3*) were higher in T1DM patients than in controls (P ≤ 0.0001). BMI values and the percentage of males were higher in control subjects compared to T1DM patients (P ≤ 0.006). Additionally, mean age at T1DM diagnosis was 17.7 ± 10.1 years, 46.7% of the cases had diabetic retinopathy (DR), and 28.8% had diabetes kidney disease (DKD) (Table 1).

**Table 1:** Characteristics of T1DM patients (cases) and nondiabetic subjects (controls) included in the study

Characteristics	Nondiabetic subjects (n = 598)	T1DM patients (n = 475)	P
Age (years)	39.8 ± 9.9	38.4 ± 13.0	0.065
Gender (% male)	59.9	51.3	0.006
Ethnicity (% black)	10.6	8.6	0.347
BMI (kg/m^2^)	27.1 ± 4.8	24.3 ± 3.8	0.0001
Hypertension (%)	5.4	39.0	0.0001
HbA1c (%)	5.3 ± 0.3	8.8 ± 2.0	0.0001
High-risk HLA DR/DQ genotypes (%)	17.3	57.1	0.0001
Age at T1DM diagnosis (years)	-	17.7 ± 10.1	-
Diabetes kidney disease (%)	-	28.8	-
Diabetic retinopathy (%)	-	46.7	-

Data are shown as mean ± standard deviation or %. BMI: body mass index; HbA1c: glycated hemoglobin. High-risk HLA DR/DQ genotypes (DR4/DQ8, DR3/DR4-DQ8 or DR3/DR3) associated with higher risk for T1DM. P-values were calculated using Student’s t test or c^2^ test, as appropriate. Only P values lower than the Bonferroni’s threshold (0.0071) were considered statistically significant.

### Genotype and allele distributions

Table 2 shows allele and genotype frequencies of the rs11755527 SNP in the *BACH2* gene in case and control groups. Genotype distributions of the rs11755527 SNP were in accordance with the frequencies estimated by the HWE in both samples (P ≥ 0.05). The frequency of the G minor allele of the *BACH2* rs11755527 SNP was 43.6% in white individuals and 37.0% in black individuals (P = 0.081). Therefore, black and white individuals were analyzed together. Genotype frequencies did not differ between patients with T1DM and nondiabetic subjects (Table 2) and this data did not change after adjustment for gender, age, BMI and high-risk *HLA DR/DQ* genotypes (Table 2). In the same way, the allele distributions of this polymorphism did not differ significantly between cases and controls (P = 0.604). Frequencies of *BACH2* rs11755527 SNP also did not differ when assuming different genetic inheritance models (dominant, recessive and additive) (P > 0.05) (Table 2).

**Table 2:** Genotype and allele frequencies of BACH2 rs11755527 SNP in patients with T1DM and nondiabetic subjects

	Nondiabetic subjects (n = 598)	T1DM patients (n = 475)	Unadjusted OR (95% CI) / P*	Adjusted OR (95% CI) / P^†^
**Genotype**				
C/C	197 (32.9)	154 (32.4)	1	1
C/G	294 (49.2)	227 (47.8)	0.988 (0.752 – 1.298) / 0.929	0.870 (0.566 – 1.336) / 0.524
G/G	107 (17.9)	94 (19.8)	1.124 (0.793 – 1.592) / 0.511	1.253 (0.731 – 2.149) / 0.412
**Allele**				
C	0.575	0.564	0.604	-
G	0.425	0.436		
**Recessive model**				
C/C+C/G	491 (82.1)	381 (80.2)	1	1
G/G	107 (17.9)	94 (19.8)	1.132 (0.832 – 1.540) / 0.429	1.367 (0.855 – 2.185) / 0.192
**Additive model**				
C/C	197 (64.8)	154 (62.1)	1	1
G/G	107 (35.2)	94 (37.9)	1.124 (0.793 – 1.592) / 0.511	1.255 (0.731 – 2.156) / 0.411
**Dominant model**				
C/C	197 (32.9)	154 (32.4)	1	1
C/G + G/G	401 (67.1)	321 (67.6)	1.024 (0.792 – 1.324)/ 0.856	0.963 (0.642 – 1.444) / 0.856

Data are shown as number (%) or proportion. *P-values were calculated using c^2^ tests (with exception of P values for genotype comparison between groups, which were obtained by univariate logistic regression analysis). ^†^ P-values and OR (95% CI) obtained using logistic regression analyses adjusting for high-risk T1DM HLA DR/DQ haplotypes, BMI, age, and gender.

According to previous studies by our group (23,24,27), high-risk T1DM *HLA DR/DQ* genotypes (DR4/DQ8, DR3/DR4-DQ8 or DR3/DR3) were associated with risk for T1DM [OR = 7.42 (95% CI 3.34 – 17.0)] in our population. However, the *BACH2* rs1175527 SNP did not influence this association [OR = 1.63 (0.48 – 6.18), obtained from the interaction analysis between the two loci], adjusting for age, gender and BMI.

Clinical and laboratory characteristics usually associated with T1DM were compared between patients broken down by the presence of the rs11755527 C/G + G/G genotypes (dominant model) (Table 3). HbA1c levels were lower in T1DM patients carrying the G allele compared to those with the C/C genotype (8.6 ± 1.9 *vs*. 9.3 ± 2.2 %; P = 0.004) after Bonferroni’s correction. The other characteristics described in Table 3 did not differ between T1DM patients with the C/C genotype and patients with the G allele (all P ≥ 0.05).

**Table 3:** Characteristics of T1DM patients broken down by the presence of the G allele of the BACH2 rs11755527 SNP (dominant model)

Characteristics	C/C (n = 154)	C/G + G/G (n = 321)	P*
Age (years)	39.5 ± 14.1	38.0 ± 12.5	0.292
Age at diagnosis (years)	18.6 ± 10.8	17.4 ± 9.7	0.322
Gender (% male)	55.6	49.2	0.230
Ethnicity (% black)	9.2	8.4	0.926
HbA1c (%)	9.3 ± 2.2	8.6 ± 1.9	0.004
BMI (kg/m^2^)	24.1 ± 3.8	24.5 ± 3.8	0.385
Systolic BP (mmHg)	124.5 ± 22.8	121.4 ± 16.9	0.201
Diastolic BP (mmHg)	79.4 ± 12.5	77.1 ± 11.3	0.084
High-risk HLA haplotypes (%)^†^	47.6	61.7	0.012
Diabetes kidney disease (%)	22.9	30.6	0.502
Diabetic retinopathy (%)	49.6	45.4	0.528

Data are shown by mean ± standard deviation or %. BMI: body mass index; BP: blood pressure; HbA1c: glycated hemoglobin. ^†^ High-risk HLA DR/DQ haplotypes: DR4/DQ8 or DR3/DR4-DQ8 or DR3/DR3. *P-values were calculated using Student’s t tests or c^2^ tests, as appropriate. Only P values lower than the Bonferroni’s threshold (0.0045) were considered statistically significant.

## DISCUSSION

BACH2 has been shown to play a key role in autoimmunity [reviewed in (9)]. *BACH2* is also important for regulation of apoptosis and inflammation in pancreatic beta-cells, suggesting an important role for this gene in development of T1DM (11). We therefore investigated the association between the rs11755527 SNP in the *BACH2* gene and T1DM. Our results suggest that this SNP is not associated with T1DM risk in our population.

GWAS and meta-analysis studies have highlighted *BACH2* among the major candidate genes for T1DM (12-15). A meta-analysis of three GWAS studies [the British Wellcome Trust Case Control Consortium – WTCCC (16), the Genetics of Kidneys in Diabetes (GoKinD) (17), and the National Institute of Mental Health (NIMH) (13)], including 1,785 American T1DM cases and 1,727 American controls, reported an association between the *BACH2* rs11755527 SNP and T1DM (P = 4.7 x 10^-12^) (13). In the same way, a meta-analysis performed by Barrett and cols. (12) also confirmed the association between the rs11755527 SNP and T1DM risk with a sample of 7,514 cases from the WTCCC (16) and the GoKind (17) studies and 9,045 controls and family sets from the Type 1 Diabetes Genetics Consortium (T1DGC).

In addition to the GWAS data, a case-control study performed in a Pakistani population reported that the minor rs11755527G allele was associated with T1DM risk (18), which is in contrast with our present data. This difference could be due to the different ethnic groups that were analyzed (Pakistani subjects *vs*. our sample of a mixed ethnicity population). Wegner and cols. (28) investigated the rs3757247 SNP of the *BACH2* gene in T1DM patients from Poland and did not find any significant association between the analyzed SNP and T1DM. In addition to the association with T1DM, other GWAS studies combined with meta-analyses have indicated that *BACH2* SNPs are associated with other autoimmune diseases such as asthma (29), Cohn’s diseases (30), coeliac disease (31), vitiligo (32), and Grave’s disease (33).

*BACH2* SNPs are associated with T1DM and other autoimmune diseases since this gene regulates T and B cell differentiation; therefore modulating autoimmune diseases by regulating the equilibrium between tolerance and immunity (8-10,34,35). *BACH2* is expressed in CD4+ and memory CD8+ T cells, and is necessary for differentiation of Foxp3+Treg cells; thus, suppressing inflammation in a Treg-dependent manner (9,10). Jin and cols. (36) demonstrated that increased *BACH2* gene expression in the peripheral blood of children positive for beta-cell autoantibodies was able to predict their progression to T1DM [HR = 3.94 (1.39 – 11.21)], consistent with the role of *BACH2* in B cell differentiation.

Moreover, Marroqui and cols. (11) showed that *BACH2* dysregulation is directly involved in T1DM pathogenesis since inhibition of this gene caused cytokine-induced apoptosis of human and rodent beta-cells through activation of the JNK1/BIM pathway and anti-apoptotic members of the BCL-2 family, whereas *BACH2* overexpression protected these cells against apoptosis (11). Hence, BACH2 seems to be a crucial factor in protection against cytokine-induced apoptosis in beta cells (11).

This study has some limitations. We cannot exclude the occurrence of population stratification bias, even though the number of black subjects was similar in case and control samples, and frequencies of the *BACH2* SNP were similar between white and black subjects. It is worth noting that adding ethnicity as a covariate in the logistic regression analyses did not change the results reported in Table 2. We also cannot rule out the possibility of a type II error when performing statistical analyses on the association between the *BACH2* SNP and T1DM. Although we had greater than 80% power (α = 0.05) to detect an OR ≥ 1.4 for T1DM risk, we cannot rule out the possibility that the *BACH2* rs11755527G allele could be associated with T1DM at lower ORs. Despite these limitations, taking into account that frequencies of the *BACH2* rs11755527G SNP are very similar between case and control groups, it seems improbable that this variant could play a major role in T1DM pathogenesis in our population. Therefore, the lack of association of the rs11755527G SNP with T1DM in our population could be explained by differences between studies in terms of the ethnicities and the genetic and environmental backgrounds of the populations studied. Our study is the first to analyze this SNP in a South American population of mixed ethnicity while previous studies mostly included North American and European populations.

In conclusion, data reported here suggest that the *BACH2* rs11755527 SNP is not associated with T1DM in our Southern Brazilian population. Additional studies with larger sample sizes are needed to better elucidate the effects of *BACH2* SNP in the pathogenesis of T1DM in different ethnicities.
